# An Intelligent Automated System for Detecting Malicious Vehicles in Intelligent Transportation Systems

**DOI:** 10.3390/s22176318

**Published:** 2022-08-23

**Authors:** Tehreem Ashfaq, Rabiya Khalid, Adamu Sani Yahaya, Sheraz Aslam, Ahmad Taher Azar, Tamim Alkhalifah, Mohamed Tounsi

**Affiliations:** 1 Department of Computer Science, COMSATS University Islamabad, Islamabad 44000, Pakistan; 2 Department of Information Technology, Bayero University Kano, Kano 700006, Nigeria; 3 Department of Electrical Engineering, Computer Engineering and Informatics, Cyprus University of Technology, 3036 Limassol, Cyprus; 4 College of Computer and Information Sciences, Prince Sultan University, Riyadh 11586, Saudi Arabia; 5 Automated Systems & Soft Computing Lab (ASSCL), Prince Sultan University, Riyadh 12435, Saudi Arabia; 6 Faculty of Computers and Artificial Intelligence, Benha University, Benha 13518, Egypt; 7 Department of Computer, College of Science and Arts in Ar Rass, Qassim University, Ar Rass 52571, Saudi Arabia

**Keywords:** certificate authority, intelligent vehicles, InterPlanetary File System, vehicular network

## Abstract

The exponential growth of intelligent vehicles(IVs) development has resulted in a complex network. As the number of IVs in a network increases, so does the number of connections. As a result, a great deal of data is generated. This complexity leads to insecure communication, traffic congestion, security, and privacy issues in vehicular networks (VNs). In addition, detecting malicious IVs, data integration, and data validation are major issues in VNs that affect network performance. A blockchain-based model for secure communication and malicious IV detection is proposed to address the above issues. In addition, this system also addresses data integration and transaction validation using an encryption scheme for secure communication. A multi-chain concept separates the legitimate and malicious data into two chains: the Integrity chain (I-chain) and Fraud chain (F-chain). This multi-chain mechanism solves the storage problem and reduces the computing power. The integration of blockchain in the proposed model provides privacy, network security, transparency, and immutability. To address the storage issue, the InterPlanetary File System (IPFS) is integrated with Certificate Authority (CA). A reputation mechanism is introduced to detect malicious IVs in the network based on ratings. This reputation mechanism is also used to prevent Sybil attack. The evaluation of the proposed work is based on the cost of smart contracts and computation time. Furthermore, two attacker models are presented to prevent the selfish mining attack and the Sybil attack. Finally, a security analysis of the proposed smart contracts with their security vulnerabilities is also presented.

## 1. Introduction

In the modern era, vehicle sectors have modernized due to advances in communication infrastructures. In recent years, the number of vehicles has also increased due to the huge population growth. This progress brings new experiences for autonomous and self-driving cars. New services are being introduced in advanced vehicles, such as communication and charging services [1,2]. The vehicle sector has also made great strides and is being transformed into a smart and intelligent network. Conventional vehicles are being transformed into smart and electric vehicles, known as electric vehicles (EVs). The EVs are connected to a network and communicate with each other. The network created by connecting vehicles and communication devices is called the Internet of Vehicles (IoVs). In IoVs, vehicles are equipped with various sensors that collect information from other vehicles and Roadside Units (RSUs) and process it for various decision-making [3,4]. They also communicate with charging stations. Vehicle to Grid (V2G) and Vehicle to Vehicle (V2V) are two common communication channels through which vehicles communicate with other units. In V2V, vehicles communicate with other vehicles and exchange information such as road and weather conditions. In V2G, on the other hand, vehicles communicate with power grids to meet their energy needs. The different communication modes for electric vehicles include Vehicle-to-Infrastructure (V2I) and Vehicle-to-Everything (V2X) [1,4]. V2I involves vehicles communicating with nearby infrastructure, while V2X involves communication between vehicles and surrounding buildings, toll booths, gas pumps, etc. Electric vehicle innovation is bringing two new concepts to market: Grid to Vehicle (G2V) and V2G [5]. EVs have bidirectional communication and energy flow. The vehicle sector has experienced rapid growth in recent years. As the number of vehicles using fuel increases, so does the likelihood of road congestion, resulting in pollution. Research and science have focused on EVs as a clean energy source for the environment. They reduce the need for oil while also reducing gas emissions.

Traditional centralized approaches used in vehicular networks (VNs) face storage and security challenges. For example, the study presented in [6] addresses model inversion attacks using deep generative models. The authors in [7] use blockchain in intelligent vehicles (IVs) for privacy and security purposes. However, the VN does not consider distributed memory management and channel reliability. Blockchain is used in the proposed system to solve security issues. It provides security to users and promotes decentralization [8,9]. It is a distributed, decentralized, and immutable ledger that provides security, trustworthiness, and transparency for data. A copy of the distributed ledger is available to all network participants.

This paper employs blockchain technology for the EV sector to solve the problem of trust between users and to ensure the immutability of data and the distinction between authentic and inauthentic data. Vehicles are validated by CA, which assigns unique identities to all vehicles. All vehicles communicate using these unique identities. When two vehicles want to communicate with each other, a smart contract is established between them for secure communication. A consensus mechanism is also used to ensure transparency. The transaction data are stored in a distributed ledger of which all nodes have a copy. The proposed work surpasses the existing work by incorporating the concept of branching the vehicles into two different branches instead of storing the data in a single blockchain. This branching mechanism also helps in reducing the computation time and storage requirement. The contributions of this work are given below:

- In the proposed work, a secure and efficient communication model based on blockchain is proposed. The proposed model addresses some main communication issues such as lack of coordination between IVs, validation of transactions, and detection of malicious IVs in a VN.
- The proposed model also helps manage the storage problem efficiently and promotes secure communication.
- CA is used for the authentication of IVs and provides trustworthiness for the communication of IVs.
- The multi-chain concept is introduced, where the integrated network entities are stored in the Legitimate Chain (L-chain), and all fraudulent entities are stored in the Fraud Chain (F-chain). In addition, the multi-chain concept also solves the intensive data problem.
- An encryption technique is used to validate the transactions (data).
- Furthermore, two attacker models, selfish mining and Sybil attack are also implemented to protect the system from blockchain attacks.

The organization of this paper is as follows. Section 2 consists of the related work and the problem statement. Section 3 discusses the proposed system model. Simulation results and discussions are presented in Section 4. Security analysis of the proposed smart contracts and attacker models are discussed in Section 5. Finally, the conclusions of the paper and future work are outlined in Section 6 and Section 7.

## 2. Related Work and Problem Statement

Blockchain is an emerging technology that is attracting tremendous attention from both industry and researchers. In this section, we discuss the current literature on blockchain in vehicular networks in detail and also elaborate these papers in Table 1.

In [10], the authors addressed the problems of security and privacy preservation through authentication in a Vehicular Ad hoc Network (VANET). Therefore, they proposed an authentication protocol for privacy preservation. However, they did not consider the storage problem because storing the authentication information of one million vehicles requires ample storage space. The privacy, authentication, and communication problems in VANETs are addressed in [11]. Therefore, a tractable decentralized framework for vehicle communication is proposed. However, the large number of events affects the efficiency of the proposed work. In [12], the authors discussed the trust mechanism in VANETs, which face various vulnerabilities, such as malicious nodes sending fake messages and trust inconsistency. Therefore, a trust mechanism with active detection has been proposed for VANETs. However, the proposed mechanism incurs a high computational cost to perform various functions. In [13], the authors dealt with the problem of deception attacks. For this purpose, an applied intelligence in blockchain VANET (ALICIA) is proposed that uses artificial neural networks (ANN). Hyperledger was also used to implement the proposed model. However, the metrics in ALICIA are lower compared to Hyperledger. In [14], the authors proposed a V2V energy trading architecture based on Fog computing to maximize social welfare (SWM). They also worked on improving Practical Byzantine Fault Tolerance (PBFT) and proposed a new consensus algorithm called Delegated Proof of Stake (DPoS). However, in DPoS, only 50% of nodes achieve the correct consensus; therefore, the efficiency of the system is compromised by other incorrect nodes. In [15], the authors propose a reputation system for intelligent transportation systems (ITS) that provides data validation for traffic data received from multiple users. However, if users do not contribute traffic data, the proposed model cannot provide information about traffic events. In [16], the authors propose a blockchain-based incentive mechanism to validate traffic events. In [17], the authors propose a blockchain-based incentive mechanism for energy trading. It enables efficient and secure energy trading between EVs and power grids. To increase the security level, they proposed a reputation model and a secure distributed energy trading system for efficient energy trading. However, malicious actors are not considered in the proposed system. In [18], the authors solved the problem of data access and authentication. In [19], the authors propose a framework for secure and efficient energy trading. The energy Internet has both information and energy flows; however, it cannot provide roaming services for vehicles. Therefore, in [20], the authors proposed an architecture that provides charging services to roaming vehicles.

In [21], the authors proposed a blockchain-based trading model for Peer to Peer (P2P) transactions among EVs. The proposed model considered the uncertainty and randomness of EV charging and discharging. In [22], the authors considered different charging infrastructures for the charging of vehicles. In [23], the authors addressed the problems of insecure communication and lack of privacy in VANETs. Therefore, they proposed an efficient mechanism for privacy preservation, aggregation of signatures, and batch verification. However, because of the high cost, it cannot maintain batch verification. In [24], the authors addressed energy trading models’ security and privacy issues. In [25], the authors addressed the trust issues and proposed a blockchain-based trusted data management scheme termed BlockTDM. The proposed scheme supports multichannel data isolation and segmentation that provides security to sensitive data. The authors in [26] proposed a mechanism to deal with the security issue of the sensors connected with vehicles. The proposed mechanism is validated through various security criteria such as fake requests, probabilistic authentication issues, etc. In [27], a blockchain-based fair non-repudiation scheme is proposed for the Industrial Internet of Things (IIoT). The authors in [28] proposed a lightweight blockchain-based model for the V2G network. The model is called Directed Acyclic Graph-based V2G network (DV2G). It deals with the issues of high computational power requirements and a lack of security and privacy. Moving ahead, the authors in [29] brought forward the concept of a novel configuration mechanism to serve the objective of deploying distributed assets. The model is designed for an automatic Frequency Restoration Reserve (aFPR) market. The authors in [30] proposed a key agreement protocol for the authentication in a blockchain-based multi Trusted Authority (TA) network.

In [31], the authors proposed a decentralized privacy-preserving scheme for EV charging. Furthermore, the energy trading mechanism is provided based on the day ahead markets by authors in [32,33]. According to the proposed scenario, a double auction mechanism is used in which all EV users have submitted their bidding price. Moreover, in [34], a secure charging scheme in a contract-based energy blockchain is proposed, which is used in smart communities. In [35], the authors proposed a blockchain-based secure charging system that resolves the security problems in vehicular systems. The proposed model is robust against the man-in-the-middle attack and replay attack. It automatically validates Internet security protocols.

In [36], the authors worked on Vehicular Social Networks (VSNs) and proposed an efficient data sharing scheme. They proposed an authentication mechanism for building trust relationships before transmitting different entities in VSN. In [37], the authors also addressed the problem of secure data storage in VNs. In [38], the authors addressed the internal and external adversarial attacks using blockchain technology. They developed a consensus protocol termed Proof of Reputation (PoR) for the security of validators. In [39], a new EV charging system based on consortium blockchain is proposed. A novel algorithm, Limited Neighbourhood Search with Memory (LNSM), is also proposed, making the contracts’ performance fast and efficient. In [40], the authors addressed how incremental robotics and association amplify the chances for a wrong person to attack the transportation system successfully. A blockchain-based framework for securing smart vehicles (B-FERL) is proposed. In [41], the authors addressed various challenges such as data security threats and privacy leakage. Therefore, they proposed a system of consortium blockchain-enabled framework. A convex-concave algorithm is used to solve the problem of contract optimization. In [42], the authors also addressed the problem of network performance optimization and secure management. Therefore, they proposed a Lighting Network Smart Contract (LNSC).

In [33], the authors proposed a new system of blockchain-based energy in smart cities for EVs. The proposed trading logic contains a mechanism for auction that can be defined and applied in smart contracts within smart cities. In [43], the authors addressed the issues of communication and insecure transactions. Therefore, they proposed adopting blockchain expertise in Real-Time Applications (RTA). It significantly overcomes the problems faced in V2X transactions. In [44], the authors suggested a reliable and smart EV transportation system developed using machine learning and blockchain. They also implemented an independent study to show the effectiveness and efficiency of the proposed method. In [45], the author proposed a new method known as Proof of Driving (PoD) to select the pool of honest miners randomly. This technique, introduced in the blockchain VANET framework, makes the PBFT settlement suitable in a large public vehicle network. In [46], the authors also used PBFT for the consensus mechanism.

The authors in [47] introduced a Secure and Highly Efficient Practical Byzantine Fault Tolerance (SG-PBFT): a stable and highly productive PBFT Internet-based vehicle consensus algorithm developed on a distributed blockchain system. The distributed architecture has reduced the burden on the central server and minimized the possibility of single-node threats.

In [48], the authors address the problem of steering actuator fault in an automated vehicle. Therefore, they proposed a model based on SVM and fault detection is performed by classification. An unbalanced training dataset is used to train the model, and this model is used to diagnose the faults.

**Table 1:** Related Work.

Addressed Problems	Proposed Solutions	Strengths	Limitations
Security and privacy preservation problems [10]	Proposed an authentication protocol for privacy preservation	Outperforms in terms of delay, throughput and packet dropping rate	Does not consider the storage problem
Privacy, authentication, and communication problems in VANETs [11]	Traceable decentralized framework is proposed	Achieved conditional privacy for anonymous vehicles	Large number of events affect the efficiency of the network
Malicious nodes broadcast forged messages and trust inconsistency [12]	An active detection trust mechanism is proposed for the VANETs	Identitify the malicious behavior of EVs	Proposed mechanism has high computational costs
Problem of illusion attacks [13]	An Applied Intelligence AppLied Intelligence in bloCkchaIn VANET (ALICIA) is proposed	Novel validation scheme	Low-level metrics in ALICIA
Inefficient Energy trading [14]	Proposed a fog computing-based V2V energy trading architecture	Improved PBFT	System’s efficiency is threatened
Centralized storage and data validation problems [15]	Proposed a reputation system for ITS	Secure data sharing	Validation of information is based on users
Storage issue [16]	Proposed system exploits benefits of IPFS	Provide monetary incentives to active EVs	Consensus mechanism uses high computational power
Inefficient energy trading and security issues [17]	Proposed an incentive scheme based on blockchain for energy trading	Enhance the security level	Distributed architecture for data storage is missing
Trust, authentication, and access control problems [18]	Multiple smart contracts are proposed	Detection of misbehavior	Difficult to manage revolutionary mobile communication
Address the problem of peak hours charges [19]	Proposed a framework for secure and efficient energy trading	Incentive mechanism for the motivation of EVs	An action to prevent against various attacks is missing
Address the charging problem of roaming vehicles [20]	Proposed an architecture that provides charging services to roaming vehicles	Fair and unified billing solution	High computational power is used
Problem of uncertainty and randomness of EV charging and discharging [21]	Proposed a blockchain-based trading model for Peer to Peer (P2P) transactions among EVs	Reduce the electricity purchaser cost	Face difficulty dealing with dense network
Growing energy demand issue in EV sector [22]	Proposed different charging strategies for EVS	Modify the load profile and reduce the cost	Work only for economic perspective
Addressed the problems of insecure communication and lack of privacy in VANETs [23]	Proposed an efficient mechanism for privacy preservation, aggregation of signature, and batch verification	Reduce the risk of privacy disclosure	However, because of the high cost, it cannot maintain the batch verification
Addressed the security and privacy issues in energy trading models [24]	An account generation technique is proposed	Provides a fault-tolerant and reliable data storage	Scalability issue when the number of EVs are increased.
Address the trust issues [25]	Proposed a blockchain-based Trusted Data Management scheme	Provides data protection and mutual authentication	Designing a uniform data format is still missing
Address the security issues [26]	Proposed a mechanism to deal with the security issue	Provides secrecy and protection to the control system	High maintenance cost
Address the repudiation issue [27]	Proposed a blockchain-based fair non-repudiation scheme	Smart contract is implemented to resolve the disputes	Reputation system for service providers is missing
High computational power requirement and privacy [28]	Proposed a lightweight blockchain-based model for V2G network	Negotiate between the vehicle and grid at less cost	Designing the blockchain layer in proposed framework is an issue
Imbalance distribution of assets [29]	A novel configuration mechanism to serve the objective of deploying distributed assets	Automation is achieved	Size of memory is not calculated
Address the authentication problem [30]	Proposed a key agreement protocol for the authentication	Reduce the time of authentication	Unable to deal with damage of data
Address the privacy problem during the charging of EVs [31]	Proposed a decentralized privacy-preserving charging scheme based on blockchain and fog computing	Blockchain is deployed on fog computing nodes	Only theoretical analysis is provided
Address the problem of privacy in sellers [32]	A double auction mechanism is proposed	Case studies are provided to show the effectiveness of proposed model	Off-chain payment can cause disputes
Security problems in vehicular systems [35]	Proposed a secure charging system based on blockchain	Energy allocation mechanism to allocate the limited renewable energy for EVs	Latency issue
Storage problem [36]	Proposed an efficient data sharing scheme	Consensus and signature mechanism guaranteed the data security	Current storage mechanisms need to be revised to handle the growing size of blockchain
Addressed the internal and external adversarial attacks [38]	Develop a consensus protocol termed as Proof of Reputation (PoR) for the security	Implemented secure energy delivery	Computationally expensive
Inefficient charging issue [39]	a new system of EV charging based on consortium blockchain is proposed	Provided convenient charging services for EVs	Scalability issue
Security threats of data and the leakage of privacy [41]	Proposed a system of consortium blockchain-enabled framework	Analyze the efficiency and security of proposed model	Computational Overhead
Issues of communication and insecure transactions [43]	Proposed adopting blockchain expertise in Real-Time Applications (RTA)	Achieved secure communication and create decentralized cloud computing platform	No assessment mechanism for unreliable source
Central server for IoVs that creates security issues [47]	Introduced a Secure and Highly Efficient Practical Byzantine Fault Tolerance (SG-PBFT)	Reduced the burden on the central server and minimized the possibility of single-node threats	Required highest Cost

### Research Gap and Problem Statement

The exponential growth of IVs has led to the construction of a complex network that complicates communication between network entities. In [49], the authors used an ITS to ensure efficient communication between IVs in a VN. In ITS, a Dedicated Short Range Communication (DSRC) is used for communication. However, this protocol does not guarantee the security of the data transmission channels. DSRC is also not able to provide scalability. This is due to the fact that this protocol cannot work efficiently when there is a high volume of traffic. In [8], the authors address user access issues in data-intensive applications. The proposed solution considers data authenticity through a consensus mechanism and a deep learning mechanism. However, authenticity through consensus requires excessive computational operations and time. Moreover, the authors did not consider illegal access to data and efficient memory usage. In [8], blockchain-based device-to-device (D2D) communication is used for security purposes. However, the dense traffic between nodes and the reliability of the channel were not considered in the proposed model. In [50], the authors addressed data authenticity and proposed an anonymous onboard network authentication protocol that provides authentication for network users. However, this protocol cannot detect malicious IVs in the network. During vehicle communication, some important issues are also addressed in the IV network, such as data accuracy and data sharing in communication channels. Security and trust are also critical issues in VNs. Industry-based blockchain technology in VNs is proposed in [51]. In [52], the authors address the problem of the intersection of IVs. Four IVs reach the intersection almost at the same time, resulting in a deadlock. They solve this problem using a consensus mechanism and a mining process. However, this process causes additional computational work, consumes a significant amount of excess power, and increases the delay.

A secure blockchain-based system is proposed that enables secure communication over blockchain to solve the problems stated above. Encryption is used for data security, and malicious IVs are detected through authentication. CA is used to register IV, while the InterPlanetary File System (IPFS) is integrated with CA to solve the storage problem. A multi-chain mechanism is also used to validate data and detect malicious IVs in the VN. Based on the reputation mechanism, an intersection scenario is also proposed. The reputation mechanism is also helpful in preventing Sybil attacks.

## 3. System Model

A blockchain-based trusted vehicular model is proposed, which is an extension of [53]. The proposed model resolves the security issues in the network and manages the coordination problem among vehicles in dense traffic. As in previous systems, DSRC is used for communication that cannot provide secure data transmission. Therefore, in the proposed system, we use an encryption scheme with a communication protocol that secures the data transmission. Moreover, the problem of limited storage capacity is solved by using IPFS. In the proposed blockchain system, a concept of branching is used, where two separate chains are used to store data. The integrity chain (I-chain) stores the authentic IVs and valid transactions, and the F-chain contains the data related to malicious IVs. The problem of intensive data and dense traffic is solved by branching. Security and trust are also critical issues in VNs, and the malicious activities of IVs are also a major problem in VNs. In addition, the registration and authentication process of IVs also make our system secure. First, all IVs are registered via CA and are assigned a unique pseudonym ID. IVs use these pseudonym IDs for further communication, e.g., V2I and V2V communication. During IVS communication, multiple transactions have taken place, and after validation, these transactions are stored in the I-chain. In addition, each IV has a unique reputation value that indicates the credibility of an IV in that particular network. We use the self-confidence factor to compute the reputation values of IVs (details are described in Section 3.5). When an IV behaves maliciously, its reputation value decreases, and after exceeding a certain threshold, the IV is declared as a malicious IV and excluded from the network. However, the details of the malicious IVs are stored in the F-chain so that these IVs do not gain access to the network again. Moreover, the overlap problem is also solved on behalf of the IVs’ reputation values (details in Section 3.6).

**Figure 1:**
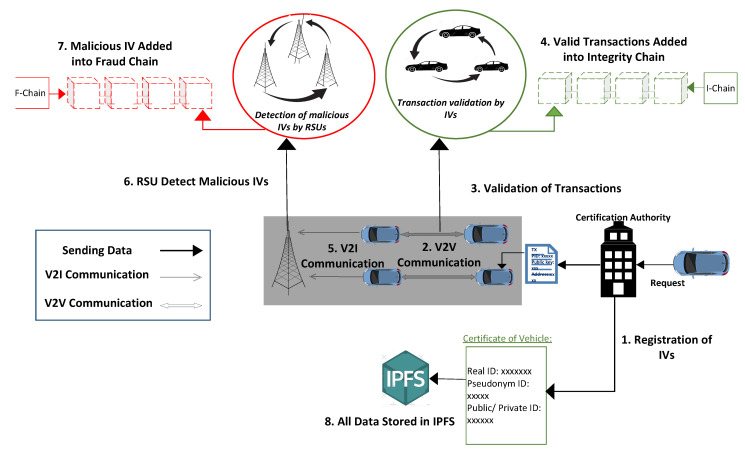
The Proposed Model for the Detection of Malicious IVs.

The proposed system model consists of four main components: IVs, RSUs, CA, and IPFS. In the proposed system, authentication between IVs is performed via CA, secure communication of IVs via blockchain, validation of transactions, detection of malicious IVs, and efficient storage management. The scenario of the proposed system is described in Figure 1.

### 3.1. Registration of IVs

**Figure 2:**
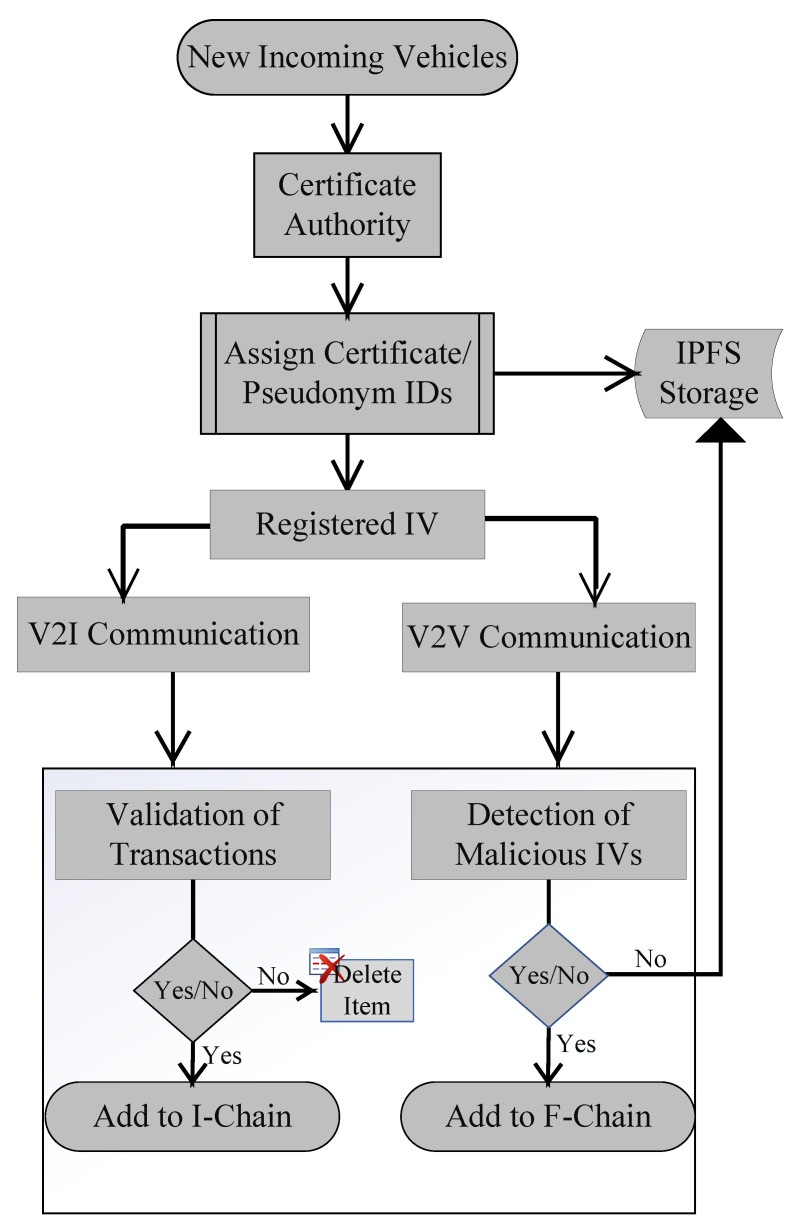
Flow Chart of the Proposed scenario.

In the modern era, the latest technologies introduced in every sphere of life are connected to the Internet, and IVs are one of them. These IVs are connected to RSUs and communicate with them, resulting in a VN. When an IV wants to join the network, it sends a request to CA and CA works as a registrar in our proposed model. It collects all relevant information about the IV and provides a digital certificate to the IV. This certificate consists of a unique ID for the IV, the pseudonym ID, the private key, and the public key. In the VN, the IVs communicate with each other using this certificate. CA is also used to authenticate data, which preserves the integrity of the data. Authentication provides data security and information assurance. For handling intensive data, authentication of data by registration is used. This process allows users to join the network based on their authenticity, which increases the efficiency and performance of the network. Figure 2 shows the scenario when a new IV wants to join the network. This figure shows IV registration, type of communication, and further processing after communication.

#### Analysis of Algorithm 1 (Registration and Validation of IVs)

In the proposed model, we use the permission blockchain. If an IV wants to join the network, it has to register. In the first step, IVs submit their information to CA to obtain a registration certificate. These are secure digital certificates that are cryptographically linked. The certification process is a one-time process where an IV interacts with CA and receives a unique pseudonym ID. For registration, the real ID and MAC address of the IV are used as input, which requires less computational power and time. However, since CA is a centralized authority, assuming that CA is a trusted authority, we can say that the certification process is secure. IVs communicate within the network using assigned pseudonym IDs, and for the first time, the validation of new IVs is also performed. All registered IVs are stored on IPFS, a decentralized storage that requires less computing power for data storage.
**Algorithm 1:** Registration and Validation of IVs.1:Initialization2:**Inputs:**  Number of IVs, MAC Address3:**Outputs:**  Registration of IV, Validate MAC Address, Stored in IPFS4:**while** IV connect with network **do**5:Registration of IV6:Check $Real_{ID},IV_{owner},MAC_{address}$7:return Registered IV8:“Validation of IV”9: **if** $hash_{1}==hash_{2}$ **then**10: “Requested IV is valid”11: **else**12: “Requested IV is invalid”13: **end if**14:“Validate MAC”15: $MAC_{1}=$AddressonIV16: $MAC_{2}=$AddressonIPFS17:**if**$MAC_{1}=MAC_{2}$**then**18: “MAC is valid. IV is registered on the network”19:**else**20: “MAC is invalid. IV is not registered on network”21:**end if**22:“Stored on IPFS”23: “Send data to IPFS”24:IPFS response25: “Return hash of data”26:**end while**27:**End**

### 3.2. Secure Communication

In smart cities, secure communication among IVs is a major concern. Therefore, an advanced encryption standard (AES) encryption scheme is used. When two IVs initiate a transaction, one IV creates a symmetric key to secure the data and sends it along with the data to the other IV. In AES, a single symmetric key is used for encryption and decryption. Once an IV shares its key with another IV, the data are exchanged between them in cipher text that is readable only by the symmetric key created by the IV. The creator IV discards the symmetric key when the transaction is complete. A new symmetric key is used for each new transaction. The AES can resist brute force attacks due to its complex and symmetric key. Therefore, it is an efficient and secure encryption technique with low computational power. A smart contract is used during the communication of the IV. The proposed smart contract avoids the involvement of a third party and solves the trust issues. In the proposed model, CA assigns unique pseudonym IDs to the vehicles. The details of each IV are stored in the IPFS.

#### Analysis of Algorithm 2 (Authentication of IVs)

As we use the permission blockchain to improve the security of the network, authentication is an essential part of network security, and it makes the network secure by allowing only registered users. CA is also used for authentication, and at the time of authentication, CA matches the pseudonym ID and the real ID of an IV with the certificate stored on the server. The IVs use the assigned pseudonym IDs for further communication. When an IV needs a service, it sends the request to RUS with the service name and the service ID. The RSU verifies the authenticity of the IV and then provides the service. After the service transactions are completed, all valid transactions are added to the I-chain. However, all malicious or inauthentic IVs are stored in the F-chain. This branching approach efficiently solves the high data volume problem and consumes less computational power.
**Algorithm 2:** Authentication of IVs.1:Initialization2:**Input:** Request Service, Real${}_{ID}$ Validation3:**Output:** Avail service, Authorization, Authentication, Validity4:**for** Authorization **do**5:Check $IV_{ID}$, PrivateKey, PublicKey, $IV_{hash}$, $User^{\prime }s$Signature6:**return** Authorized7:Authentication8: **if** ($Real_{ID}$== $New_{ID}$) **then**9:  “IV is authentic”10: **else**11:  “IV is unauthentic”12: **end if**13: **for** Request Service **do**14:“Match the service IDs”15: Req_Service[ser_id].ser_name=ser_name16: Req_Service[ser_id].ser_id=ser_id17:**return** Service18: **for** Add Service **do**19: serv[service_id]=service(service_id,20: service_name,serv_provider,serv_reciever)21:**return** Service Added22: **for** Data added into the I-Chain **do**23: transaction=valid24: **return** “Add transaction into I-Chain”25: **for** Add malicious IVs into F-Chain **do**26: IV=Malicious27: **return** “Added into F-Chain”28:**end for**29:**end for**30:**end for**31:**End**

### 3.3. Efficient Storage Management through IPFS

Efficient storage management is an essential problem in VN, which is solved by the IPFS. It is a distributed P2P network used for storing and sharing data. It stores data by its hashes, and these hashes are stored on the blockchain and mapped with a distributed hash table (DHT). When data are stored on IPFS, it is divided into chunks, and each chunk contains 256 Kbs. The hash value of each chunk is calculated and updated in the DHT and stored on the blockchain. The DHT provides decentralized and autonomous storage of hashes and makes the system fault-tolerant and scalable. The same DHT is also used to calculate the reputation values of the IVs. Algorithm 1 shows data validation and storage on IPFS. The data related to IVs are stored on IPFS, and the hashes of the stored data are uploaded to the I-chain or F-chain.

### 3.4. Branching of Data

The number of IVs has increased rapidly, creating a complex network. As the number of IVs increases, the data associated with the IVs also increases and becomes more extensive. In previous systems, the data of IVs and their transactions are stored in the same blockchain. It becomes challenging to deal with such data from the user’s perspective. Therefore, we divide the data of the transactions of the IVs and the IVs into two chains: the I-chain and F-chain. The transactions between IVs are validated by encryption and stored in the I-chain. Algorithm 2 shows the validation of the data. Data are shared between IVs in a secure and decentralized P2P manner. Users of IVs share data only with registered and authenticated IVs. When a new, unrecognized vehicle requests to share data, it must first register and obtain a certificate from CA. Sharing data in a secured and trusted environment ensures the integrity of the data and the removal of fake data spread by malicious vehicles.

### 3.5. Detection of Malicious IVs

The detection of malicious IVs in the VN is a major concern, and a reputation mechanism (Eigen trust factor) is used to detect the malicious IVs [54]. In the proposed networks, each IV has limited interaction with other IVs because there are two communication modes. Therefore, the first advantage is that there is no need to process intensive data, and the second is that the number of forwarded messages is lower. This means that each IV can report directly on other IVs. The following Equation (1) is used to calculate the self-confidence factor:

(1) $$t_{i}^{\left(k+1\right)}=\left(1-a\right)\left(C_{1i}t_{1}^{K}+C_{2i}t_{2}^{K}+...+C_{ni}t_{n}^{K}+ap_{i}\right)$$

where *i* is any random IV, t is the trust value, k is the peer of i, c denotes as the matrix, pi is the set of peers, and a is a constant less than 1. We can also say that “a” is known as a threshold value. In the proposed network, IVs communicate with RSUs to share data. IVs send data to RSUs related to road conditions, weather conditions, and traffic information. If an IV sends malicious data or incorrect information, its reputation goes down in the network, where reputation shows the credibility of an IV. When the reputation of an IV becomes less than the threshold value, it is declared as a malicious IV and added to the F-chain. Algorithm 2 shows the detection of malicious IVs.

### 3.6. Intersection Scenario

**Figure 3:**
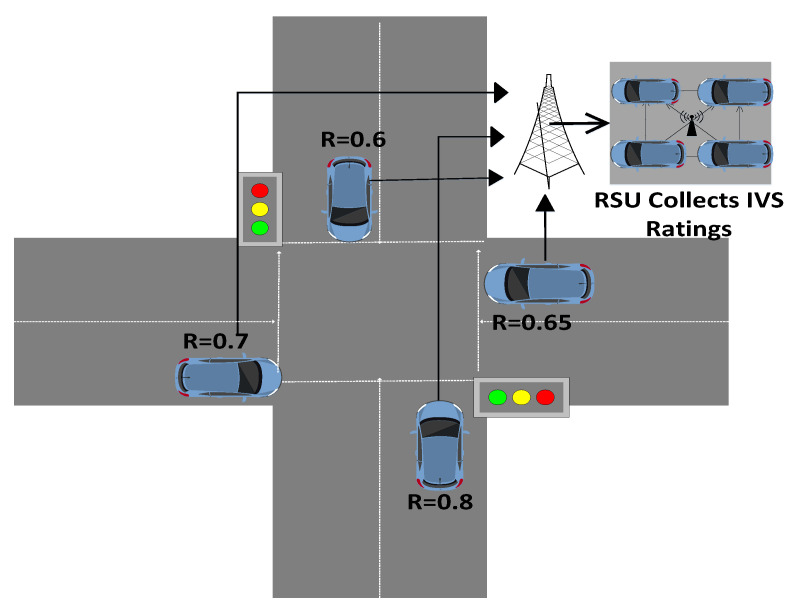
Intersection Scenario.

In this scenario, four IVs are mentioned in Figure 3 as IV1, IV2, IV3, and IV4. When they reach the intersection, all four IVs send messages to the RSU about their reputation value in the network. Then the RSU compares the reputation value of all IVs and assigns priority to the IV with the highest reputation value to move first. All IVs receive the message from the RSU according to their reputation value. In the initial phase, all IVs have the same position. The priority of the vehicles is changed according to the reputation values, and these reputation values are assigned by using Algorithm 3. The vehicle with the highest reputation value gets the chance to move first.
**Algorithm 3:** Assign Reputation to IVs.1:Initialization2:**Inputs:**  Number of IVs, Reputation value, Service3:**Output:**  Number of valid IVs, Reputation status, Service provided4:**for** the number of IVs **do**5:Check IDs6: **if** ID,owner,registrationstatus==Valid **then**7: Calculate the total number of valid IVs8: **else**9: Break10: **end if**11:**end for**12:**for** the validated IVs **do**13:“Assign reputation”14: **if** IV == valid **then**15: Increase reputation value16: **else**17: Decrease reputation value18: **end if**19:**end for**20:**for** Registered IVs, check for service **do**21: **if** service request found **then**22: Provide service23: **else**24: Deny service25: **end if**26:**end for**27:**End**

## 4. Results and Discussion

The simulation results and their discussion are presented in this section. Smart contracts are proposed to ensure the validation of the proposed system. The execution and transaction costs are used to evaluate the performance of a smart contract. These smart contracts are deployed on Remix IDE (online platform), and MetaMask is used for transaction validation [55].

**Figure 4:**
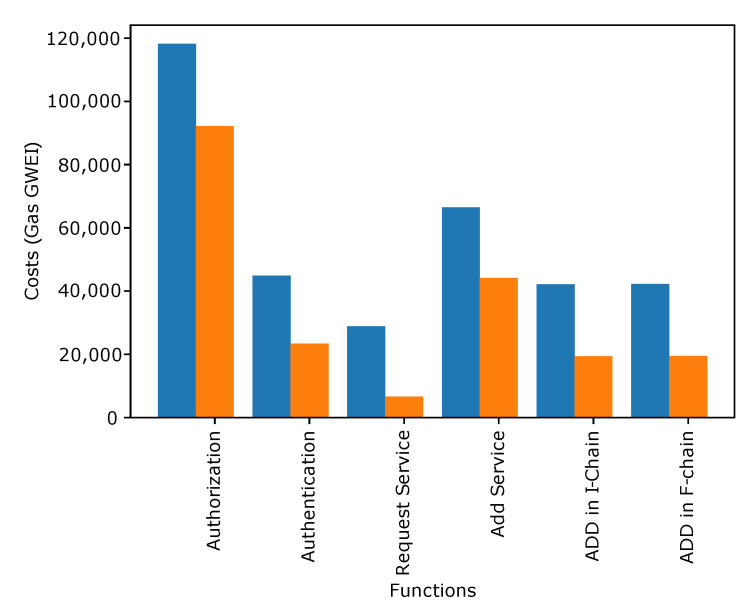
Authentication of IVs.

**Figure 5:**
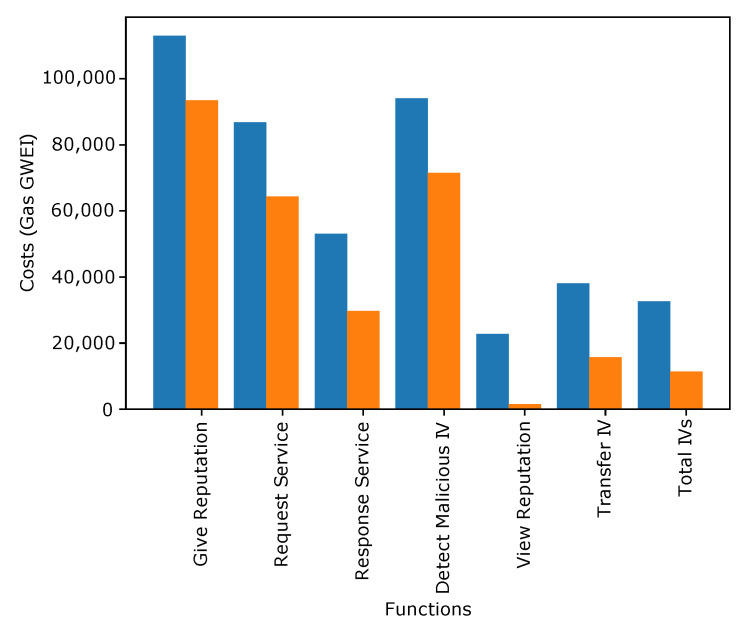
Assign Reputation to IVs.

**Figure 6:**
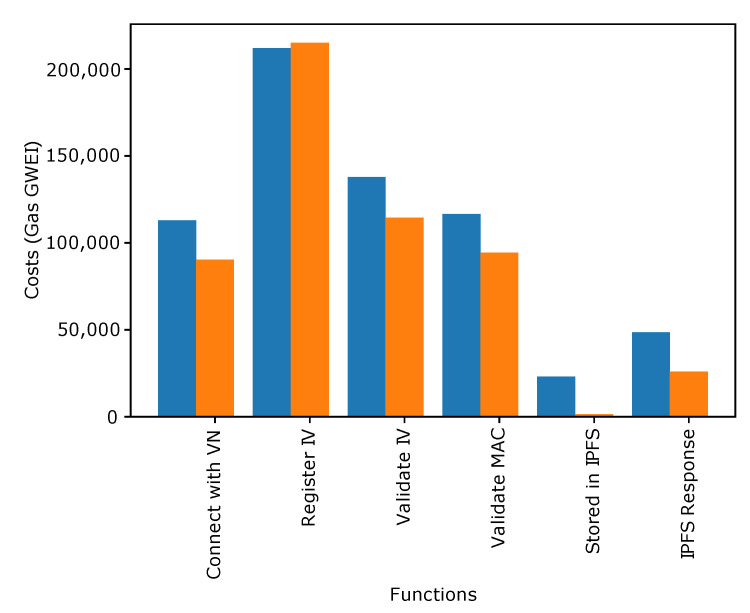
Data Stored in IPFS.

Figure 4, Figure 5 and Figure 6 show the transaction and execution costs for the smart contracts and the functions deployed in them, in terms of gas. These values are taken from RemixIDE. Fluctuations can be observed in the gas values for different functions and the contract deployment cost of these contracts are shown in Table 2, Table 3 and Table 4.

Figure 4 shows the transaction and execution cost in GWEI for different functions involved in the smart contract. The functions included in this figure consist of *‘Authorization’, ‘Authentication of IVs’, ‘Add Service’, Request Service, ‘Add in I-Chain’, and ‘Add in F-Chain’*. It is observed from the figure that the authorization of IVs has the maximum cost compared to other functions because different parameters are counted at the time of authorization.

In the blockchain, the gas consumption cost for different functions performed while giving reputation to IVs is given in Figure 5. The values are given for transaction and execution costs. The functions included in the registration process consist of a service request and response, detecting malicious IVs, giving reputation, etc. The reputation is provided upon successful service provisioning. It is visualized from the figure that the maximum cost is incurred when giving a reputation to IVs.

Figure 6 shows that the blockchain gas consumption cost is given for different functions in GWEI. The values are given for transaction costs and execution costs.

The functions for which the gas consumption values are given include the registration and validation of IVs, validation of MAC address, data storage, and response by IPFS. It is observed from the figure that the maximum cost is for the registration of the new IVs. It is because different features are included when performing IV registration.

**Figure 7:**
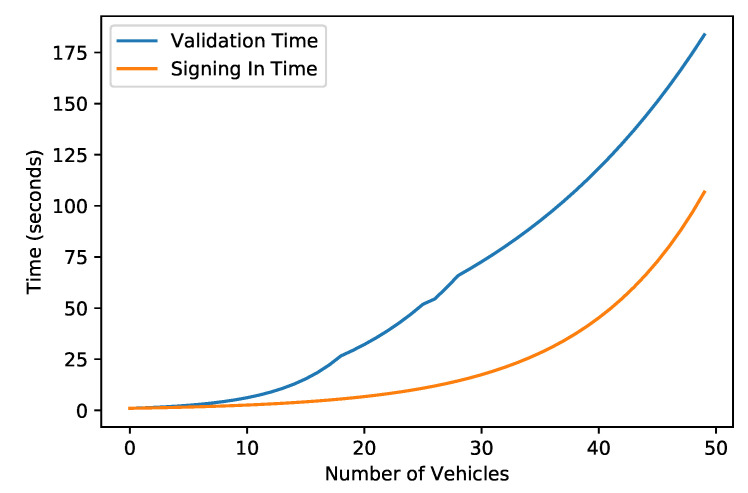
Validating and Signing Time against the Number of Vehicles.

Figure 7 shows the time taken for the signing and validating processes. It depicts that the signing-in process takes longer than the validation process. When an IV enters the network for the first time after registration, it needs to be signed in. On the other hand, when an IV performs any transaction within the network, it needs to be validated. However, the processing time increased with an increase in transaction numbers. Figure 7 shows a linear growth with an increase in the transaction number.

**Figure 8:**
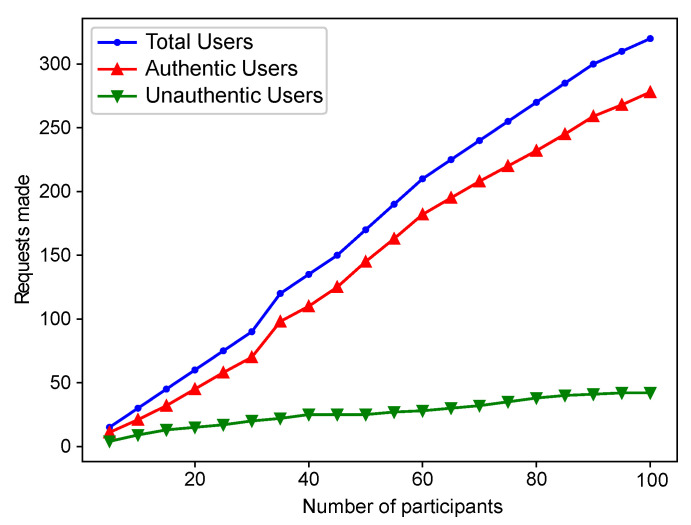
Users’ Status in Network.

Figure 8 shows the total users and requests generated by the users. It also shows the authentic users and unauthentic users in the network. According to the proposed scheme, authentic users are added to the I-Chain, and unauthentic users are part of the F-Chain. This graph shows the number of user requests (IVs). When the total data are split into two parts, it becomes easy to deal with it and respond to a large number of requests in less time and with less delay.

**Figure 9:**
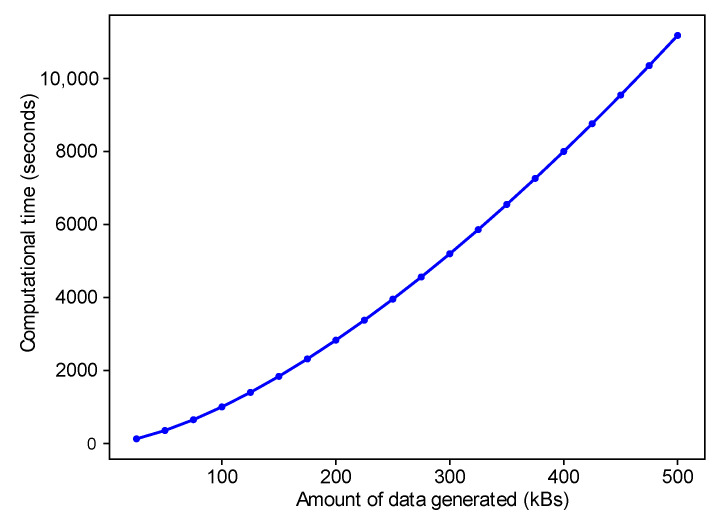
The Generated Data against Computational Time.

Figure 9 shows an exponential trend; when the amount of data increases, the computational time to process the data is also increased. It is directly related to the number of IVs in the network because when the number of IVs increasesm, the data related to these IVs also increases.

**Figure 10:**
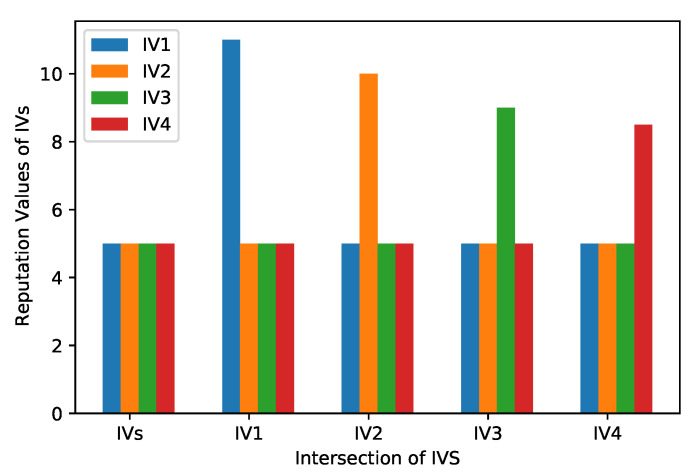
Intersection Scenario of IVs.

Figure 10 shows that if four IVs reach near crossroads approximately simultaneously, it creates a deadlock. In this figure, four IVs are mentioned as IV1, IV2, IV3, and IV4. These IVs are connected with the RSUs and share their location, speed, and reputation values. Therefore, when IVs reach the intersection point, RSU allows the IV with the highest reputation value to cross the intersection first. Afterward, the same pattern is used for other IVs. In the proposed scenario, IV1 moves first because of its highest reputation value. After IV1 is passed, IV2 and IV3 pass. When these IVs have crossed the intersection junction, IV4, with the lowest reputation value, gets the signal that the road is free. Figure 10 depicts IVs’ scenario to avoid the deadlock.

In the following Table 5, all limitations are mapped with proposed solutions.

## 5. Security Analysis

In this section, we will discuss several smart contract-based attacks and blockchain-based attacks handled by our proposed system.

### 5.1. Smart Contract-Based Attacks

**Figure 11:**
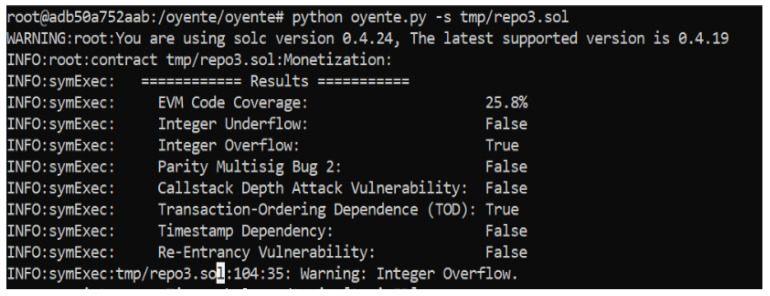
Security Analysis of the Proposed System.

**Figure 12:**
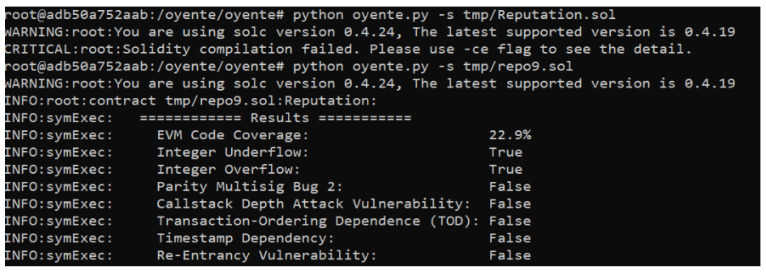
Security Analysis of the Proposed System.

**Figure 13:**
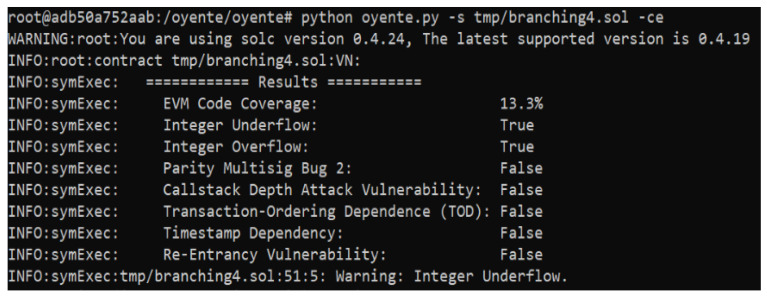
Security Analysis of the Proposed Smart Contracts.

Three smart contracts are proposed, which are susceptible to some attacks and vulnerabilities. Therefore, a vulnerability analysis is performed for our proposed smart contracts. It is essential to ensure that these contacts are bug-free and error-free because of the involvement of monetary transactions. Oyenete, an open-source tool, is used to analyze the vulnerabilities of smart contracts [56]. The working of Oyente is dependent on Ethereum Virtual Machine (EVM), and solidity repository Solc [57]. Multiple attacks are reported during the analysis, such as Re-Entrancy Vulnerability, Timestamp Dependency, Transaction-Ordering Dependence, Parity Multising Bug 2, and Callstack Depth attack. The analysis of the proposed smart contracts is represented in Figure 11, Figure 12 and Figure 13.

#### 5.1.1. Types of Smart Contract Attacks

Several smart contract-based attacks are discussed below.

**Re-Entrancy Attack:** In this attack, the attacker takes over the control flow of a smart contract. However, in the current Ethereum chain, this security vulnerability has not existed.

**Timestamp Dependency:** This vulnerability is created when a miner manipulates the timestamp of a block to generate their desired output. It is a miner-centric attack that is initiated by a participating miner.

**CallStack Depth Vulnerability:** According to this attack, if the call depth of a function is equal to 1024 frames, the calling function only works until 1023 frames, and the call may fail. An attacker might be able to launch this attack if they force the call stack to the maximum value.

**Transaction Ordering Dependency:** In this vulnerability, the attacker can easily manipulate the gas prices and order of transactions. This attack can manipulate all dependent transactions.

**Integer Overflow and Underflow:** The integer overflow occurs when the incremental value exceeds the fixed threshold limit. On the other hand, integer underflow occurs when the value decreases from the fixed threshold value.

#### 5.1.2. Security Features

In this subsection, we discuss the security features of our smart contracts and how our system ensures security against security attacks. These features are integrity, decentralization, non-repudiation, trust, and availability. This system is protected against re-entry attacks, call stack depth attacks, etc.

**Integrity:**This feature ensures data integrity, and any other entity does not modify that data. The immutability of blockchain also helps to overcome the issue of data modification and store all the data for a long time.

**Availability:**This feature ensures that all smart contracts in the blockchain must be available for all participants. It also provides service availability for participants. It protects the system from Denial of Service (DoS) attacks. Moreover, a blockchain ledger is highly robust against DoS attacks.

**Confidentiality:**The confidentiality of a system is about protecting a system’s data against unauthorized and unintentional access. It also maintains the privacy of the system. The confidentiality of our system is achieved through a permissioned blockchain.

### 5.2. Blockchain-Based Attacks

In this section, we discuss various blockchain-based attacks that are defended against by our proposed system in detail. We consider selfish mining and Sybil attacks since the probability of occurrence of these two attacks is higher in the proposed model. If we protect our system from these two attacks, other related attacks cannot damage the proposed system.

#### 5.2.1. Selfish Mining Attack

Various attacks such as DoS attacks, Sybil attacks, and double-spending attacks are carried out in blockchain networks. One of them is the selfish mining attack, where the miner keeps the block for a certain time before releasing it when the stakes are high to get the most value [58].

Two parameters, α and γ, are crucial in a selfish mining attack. The first parameter indicates the probability of the attack when a malicious node forces the honest nodes to add the F-chain to the network. The other symbol, on the other hand, indicates the probability of when a selfish node takes over the blockchain.

(1−α) and (1−γ) reflect the mining power and the probability of an honest miner, respectively. Both parameters’ values are in the range of 0 to 1 [59].

According to the literature, when a selfish node’s mining capacity, i.e., α, crosses a certain threshold, the selfish node assumes control of the entire network and forges it according to its desires. As a result, the $\frac{1}{3}$ threshold value is chosen. If this value is exceeded, the greedy node begins to deviate from the predetermined protocol to maximize its income. According to [58], the selfish miner’s income falls within the range of Equation (2).

(2) $$\frac{1-\gamma }{3-2\gamma }\;<\;\alpha \;<\;\frac{1}{2}$$

Minimum and maximum values of γ are used in Equation (2); we get 0.0098 and 0.33 as the contribution of selfish mining capacity, respectively. Furthermore, as the value of α rises above 50%, the profit of the selfish miner approaches 100%. It also leads the whole network towards the 51% attack.

Different facets of the selfish mining attack include the probability of an attack, the estimation of overall profit made, and the profit and loss ratio, all listed below.

- **Probability of occurrence of attack:**
  The probability of a selfish mining attack depends on several variables, including computing capacity and selfish mining power. During the attack, orphan blocks are generated, indicating that the attack has occurred. As the probability of selfish mining increases, the number of orphan blocks also increases. The high number of orphan blocks is used as evidence of the existence of selfish miners. Simulation results show the proportional relationship between orphan blocks and the probability of attack.
- **Total revenue calculation:**
  Selfish miners in the network initially create forks in the blockchain and connect the fake blocks in the blockchain. These fake blocks get control of the network and gain revenue.
  In a selfish mining attack, selfish miners succeed in persuading the honest miners to create blocks that are then attached to the blockchain. As a result, honest miners waste resources. The revenue is measured as a percentage of the proposed work using Equation (3) and (4).
  (3) $$R_{selfish}=\frac{r_{selfish}}{r_{selfish}+r_{honest}}$$
  (4) $$Revenue=\frac{Number\;of\;selfish\;blocks\;mined}{Total\;number\;of\;blocks\;mined}*100$$
- **Profit and loss ratio:**
  The selfish mining attack is calculated using the network’s Profit and Loss ratio, abbreviated as P2LR. As seen in Equation (5) [60], P2LR is determined by subtracting the expense of $Mining_{cost}$ from the overall revenue Revenue per unit time $Time_{unit}$.
  (5) $$P2LR=\frac{Revenue-Mining_{cost}}{Time_{unit}}$$

**Figure 14:**
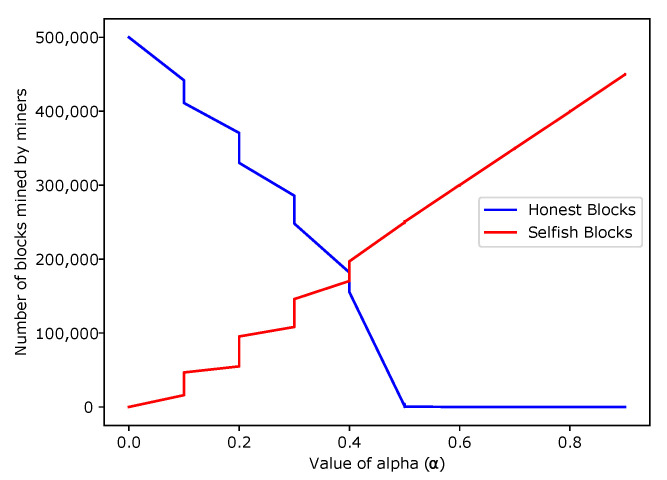
The Impact of alpha on the Number of Blocks Mined.

In Figure 14, the number of blocks mined by the honest and the selfish miners are shown. The figure shows that when the value of α increased, the number of selfish blocks also increased, whereas the number of honest blocks decreased. The increasing and decreasing trends are the increase in both the mining power of the attacker and the selfish mining attack. The blue line shows the decreasing trend of the honest miners, while the red line shows the increasing trend of the greedy miners. It is also observed that as soon as the value of α crosses 0.5, the attacker entirely takes over the network. It results in the creation of only the fake blocks.

**Figure 15:**
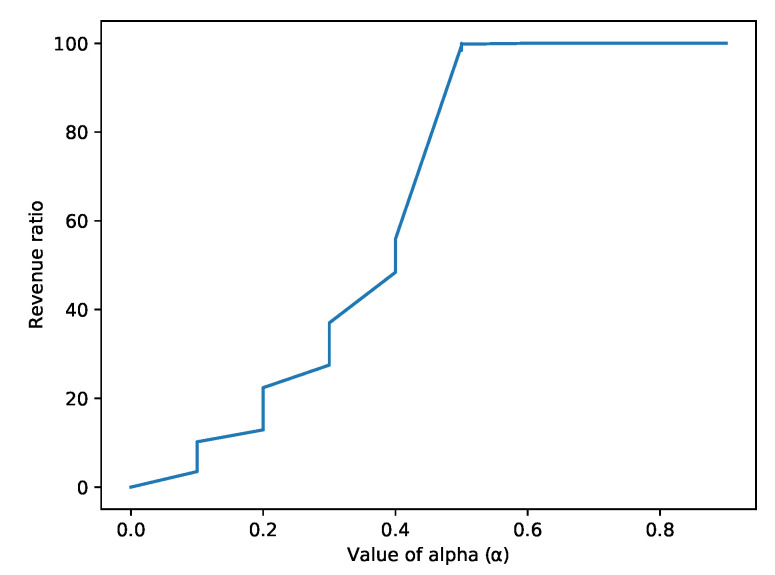
The values of alpha against the Revenue Ratio.

Figure 15 shows the revenue generated by the attackers in accordance with the α. From the figure, it is observed that as the mining power of the attacker increases, the revenue also increases. The network’s revenue becomes maximum when the value of α increases to more than 0.5. It shows that the network is robust till the value of α is less than 0.5. Once it crosses 0.5, the entire network is collapsed and is taken over by the attacker. Therefore, the robustness of the network depends on the α that works as a threshold value.

#### 5.2.2. Sybil Attack

In the Sybil attack, the attacker creates multiple fake IDs to gain control of the network. These fake IDs fool the honest nodes and get high ratings in the network. The attacker can use these ratings to get incentives from the network. In the proposed system, we introduced a reputation mechanism to solve the Sybil attack problem. In the proposed system, an EV is added to the network after registration, and a certain reputation value is assigned to the EV at the time of registration. Thus, if an EV acts maliciously and creates a fake ID, it will not contain a reputation value and will be detected as a malicious entity.

In [61], the authors discussed the idea of Sybil attack. In this attacker model, the probability of a Sybil attack is measured by various parameters such as the computational power, the number of honest nodes, and the number of fake IDs. The probability of a Sybil attack increases when the number of fake IDs increases while the computational power also increases. The following parameters are used for the Sybil attack.

(6) P(w)=mcN∗−nc−mN∗c

(7) P(w)=mnN−1N∗−nm+n−1n∗

- N: number of population
- M: number of successful items from the population
- n: number of successful items from the sample
- c: computational power of sample
- N*: number of items in the sample

**Figure 16:**
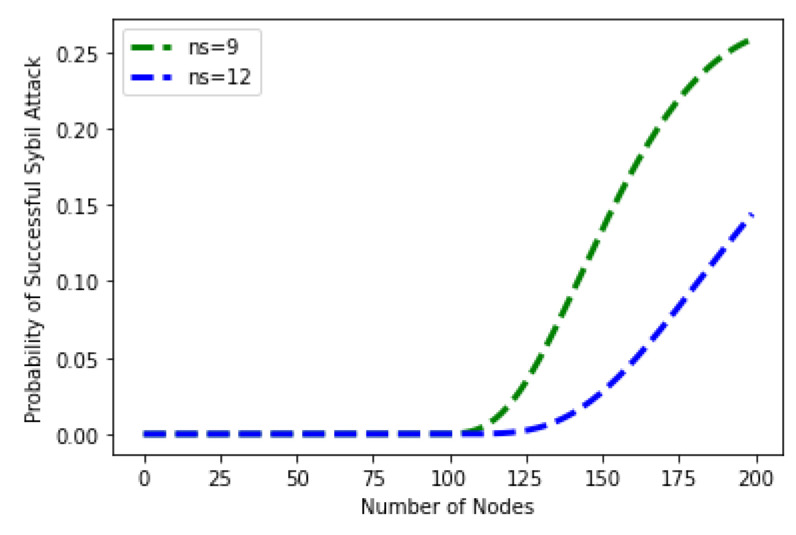
Probability of Sybil Attack versus Number of Fake Identities.

Figure 16 shows the probability of the Sybil attack by the attacker as the computational power increases. There are 200 nodes in the network, while the number of Sybil identities, i.e., the originator of the fake identity, is 9 and 12. The number of fake identities is 9, and the probability of a Sybil attack is initially zero, which increases as the computational power increases from 100. On the other hand, when the number of fake identity creators is 12, the probability of a Sybil attack increases to 125. The results in both cases show that as the number of fake IDs increases, the probability of a Sybil attack also increases. The green line is for 9 fake IDs, while the blue line is for 12 fake IDs.

**Figure 17:**
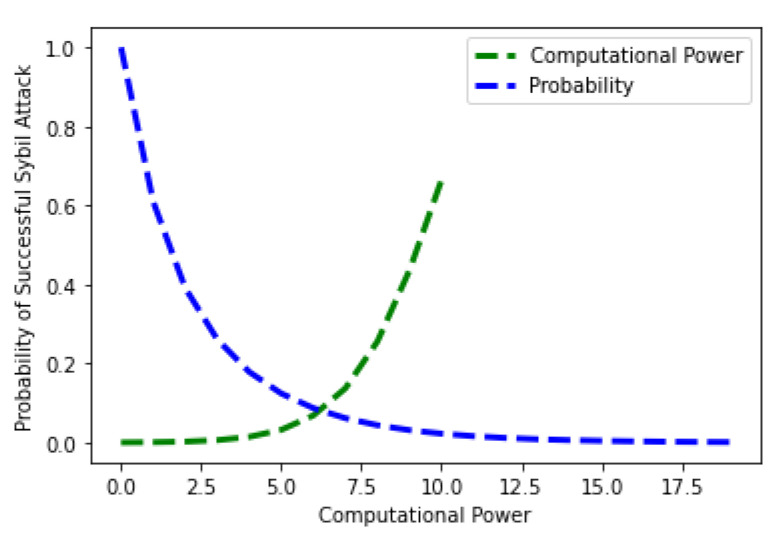
Probability of Sybil Attack versus Computational Power.

Equation (6) shows the mathematical formulation of the probability of the Sybil attack’s success. Figure 17 shows the probability of a successful Sybil attack in terms of the computational power of the honest node. It is observed from the figure that the probability of a Sybil attack is highest when the computational power of the honest node is lower. This is because no honest node is involved in the network at that time. As the honest node’s computational power increases, the probability of a successful attack decreases. The green line shows the computational power, while the blue line shows the probability of success of the Sybil attack. The mathematical formulation of the success probability of the Sybil attack in relation to the computing power is given in Equation (7).

## 6. Conclusions

In the proposed work, blockchain is used in the vehicular sector to solve security and privacy issues. The proposed model also solves the trust issues between IVs and distinguishes between authentic and inauthentic users by detecting the malicious IVs in the network. When an IV is entered into the network, it is registered through CA and gets a pseudonym ID. This ID is used for communication. In the proposed model, V2V and V2I communications are initiated, where all transactions are validated via AES and malicious IVs are detected based on their reputation values. These reputation values are generated by an intelligent contract based on the transactional history of the IVs. It also introduces a multi-chain concept where transaction data and malicious IVs are stored in two branches: the I-chain and F-chain. A smart contract is proposed for the multi-chain mechanism to reduce computation time and manage storage requirements. IPFS is integrated with CA to solve the storage problem. In IPFS, data are divided into chunks, and each chunk is assigned a unique hash value. These hash values are stored in the blockchain, and the data are stored in IPFS, which has less cost. The proposed work also solves the problem of overlapping IVs on roads. In addition, two attacker models and smart contract analysis are implemented to protect the system against bugs and attacks. The proposed system performed well against selfish mining and Sybil attack. The simulation results show that the proposed work outperforms the current work in terms of security and adequately solves the major problems of IVs.

## 7. Future Work

In this research, we have addressed the detection of malicious IVs and insecure communication of IVs and tackled the cybersecurity challenges related to blockchain security. However, there are still some gaps that need to be investigated in the future. In the future, we plan to work on inversion attacks and vehicle fault detection. In addition, we will work on network optimization.

## Figures and Tables

**Table 2:** Contract Deployment Cost of Authentication of IVs.

Parameter	Value
status	true Transaction mined and execution succeed
transaction hash	0xa4f56,…,b10edd1985594
from	0x5B38D,…,eddC4
to	VN_Authentication.(constructor)
gas	2507800 gas
transaction cost	2180695 gas
execution cost	2180695 gas
hash	0xa4f5,…,d1985594
input	0x608…10032
decoded input	{}
decoded output	-
logs	[]
value	0 wei

**Table 3:** Contract Deployment Cost of Assigning Reputation.

Parameter	Value
status	true Transaction mined and execution succeed
transaction hash	0xe19a,…,dee9d
from	0x5B38,…,dC4
to	Reputation.(constructor)
gas	477940 gas
transaction cost	415600 gas
execution cost	415600 gas
input	0x608…30029
decoded input	{}
decoded output	-
logs	[]
value	0 wei

**Table 4:** Contract Deployment Cost of IPFS Storage.

Parameter	Value
status	true Transaction mined and execution succeed
transaction hash	0x5eac1f9ea,…,71b6e139e
from	0xca35b, …, a733c
to	Storage.(constructor)
gas	144529 gas
transaction cost	125677 gas
execution cost	125677 gas
hash	0x98933,…, 75f15
input	0x60806,…,70033
decoded input	{ }
decoded output	-
logs	[]
value	0 wei

**Table 5:** Mapping Table of Limitations and Proposed Solutions.

Limitations	Proposed Solutions	Validations
Intensive data increase the computational power and delay	Division of data into multiple chains	Multi-chains are shown in terms of the relationship between the amount of data generated and time taken, as shown in Figure 9
Insecure communication	Blockchain, CA, authentication process, and AES are used to provide secure communication in the proposed system	Figure 7 shows the relationship between authentic and unauthentic users.
Inefficient storage management	Through IPFS, data are not stored on blockchain, only hashes of data are stored on blockchain	Figure 6 shows the results of IPFS storage
Road congestion	To tackle the road congestion, intersection criteria are set and followed	Intersection criteria are shown in Figure 10
Validation of transactions	The encryption technique AES is used for the validation of data	Figure 9 shows the computational time used for encryption

## Data Availability

Not applicable.

## Abbreviations

AI	Artificial Intelligent
AES	Advanced Encryption Standard
aFPR	automatic Frequency Restoration Reserve
ALICIA	AppLied Intelligence in bloCkchaIn VANET
ANN	Artificial Neural Networks
BMS	Battery Management system
BlockTDM	Blockchain-based Trusted Data Management scheme
CA	Certificate Authority
D2D	Device to Device
DHT	Distributed Hash Table
DSRC	Dedicated Short Range Communication
DPoS	Delegate Proof of Stake
EVs	Electric Vehicles
F-Chain	Fraud Chain
IDs	Identifications
I-Chain	Integrity Chain
IIoT	Industrial Internet of Things
IoT	Internet of Things
IoVs	Internet of Vehicles
IPFS	InterPlanetary File System
IVs	Intelligent Vehicles
ITS	Intelligent Transport System
LDB	Locally Dynamic Blockchain
LNSC	Lighting Network Smart Contract
PBFT	Practical Byzantine Fault Tolerance
P2P	Peer to Peer
PoD	Proof of Driving
PoR	Proof of Reputation
RSUs	Roadside Units
SG-PBFT	secure and highly efficient PBFT
SDN	Software Defined Network
SWM	Social Welfare Maximization
TA	Trusted Authority
VANET	Vehicular Ad hoc Networks
VEN	Vehicular Energy Network
VSN	Vehicular Social Networks
V2G	Vehicle to Grid
V2I	Vehicle to Infrastructure
V2V	Vehicle to Vehicle
V2X	Vehicle to Everything
VN	Vehicular Network
